# Evaluation of outcome reporting trends for femoroacetabular impingement syndrome- a systematic review

**DOI:** 10.1186/s40634-021-00351-0

**Published:** 2021-04-23

**Authors:** Ida Lindman, Sarantos Nikou, Axel Öhlin, Eric Hamrin Senorski, Olufemi Ayeni, Jon Karlsson, Mikael Sansone

**Affiliations:** 1 Department of Orthopaedics, Institute of Clinical Sciences, Sahlgrenska Academy, University of Gothenburg, 413 45 Gothenburg, Sweden; 2 Department of Orthopaedic Surgery, South Älvsborg Hospital, 501 82 Borås, Sweden; 3 Department of Health and Rehabilitation, Institute of Neuroscience and Physiology, Sahlgrenska Academy, University of Gothenburg, Gothenburg, Sweden; 4 Division of Orthopaedic Surgery, McMaster University, Hamilton, ON L8N 3Z5 Canada

**Keywords:** Femoroacetabular impingement syndrome, FAIS, Patient-reported outcome measures, PROM, Hip arthroscopy

## Abstract

**Purpose:**

The aim of this systematic review was to evaluate the trends in the literature regarding surgical treatment for femoroacetabular impingement syndrome (FAIS) and to present which patient-reported outcome-measures (PROMs) and surgical approaches are included.

**Methods:**

This systematic review was conducted with the PRISMA guidelines. The literature search was performed on PubMed and Embase, covering studies from 1999 to 2020. Inclusion criteria were clinical studies with surgical treatment for FAIS, the use of PROMs as evaluation tool and studies in English. Exclusion criteria were studies with patients < 18 years, cohorts with < 8 patients, studies with primarily purpose to evaluate other diagnoses than FAIS and studies with radiographs as only outcomes without using PROMs. Data extracted were author, year, surgical intervention, type of study, level of evidence, demographics of included patients, and PROMs.

**Results:**

The initial search yielded 2,559 studies, of which 196 were included. There was an increase of 2,043% in the number of studies from the first to the last five years (2004–2008)—(2016–2020). There were 135 (69%) retrospective, 55 (28%) prospective and 6 (3%) Randomized Controlled Trials. Level of evidence ranged from I-IV where Level III was most common (44%). More than half of the studies (58%) originated from USA. Arthroscopic surgery was the most common surgical treatment (85%). Mean follow-up was 27.0 months (± 17 SD), (range 1.5–120 months). Between 1–10 PROMs were included, and the modified Harris Hip Score (mHHS) was most commonly used (61%).

**Conclusion:**

There has been a continuous increase in the number of published studies regarding FAIS with the majority evaluating arthroscopic surgery. The mHHS remains being the most commonly used PROM.

## Introduction

In 1936 Smith-Petersen described hip pain caused by a bone-to-bone impingement between the femoral neck and the acetabulum [196]. However, it was not until 2003 that the modern concept of femoroacetabular impingement was initiated by Ganz et al. [74].

Femoroacetabular impingement syndrome (FAIS) results from an abnormal morphology of either the femoral head (cam) or the acetabulum (pincer) or a combination of both. This causes an incongruence in the hip joint and is a common source of hip pain, especially in the young active population [216]. Surgical treatment of FAIS aims to restore the normal hip joint morphology and thereby reduce symptoms [154]. Open hip dislocation was initially considered the gold standard for surgical treatment of FAIS, however, the use of a minimally invasive approach with arthroscopy has increased during the 2010′s [46, 154].

With an escalation of the arthroscopic procedures performed, there has been a corresponding increase in the studies published regarding FAIS [106]. Furthermore, several registries have been developed to keep track of performed arthroscopies and evaluate the outcomes after the procedures [93, 126, 185]. Patient-reported outcome measures (PROMs) are commonly used for evaluating the patients’ perspective of outcome of surgical treatment [158]. According to the Warwick Agreement, defined in 2016, the Hip and Groin outcome score (HAGOS) [205], Hip Outcome Score (HOS) [134] and the international Hip Outcome Tool (iHOT) [84, 143] are recommended as preferable PROMs for evaluating the outcome after FAIS surgery [82]. These PROMs are noted to be valid, reliable and responsive after FAIS surgery [170]. Yet, the PROMs used for FAIS have most commonly been developed for an older patient category with osteoarthritis, such as Harris hip score (HHS), while the PROMs recommended for the younger population are gradually being adopted [206]. With the use of PROMs developed for another patient category or condition, there is a risk of ceiling or wash-out effects due to the inclusion of non-relevant items.

The aim of this systematic review was to evaluate the trends in the literature pertaining to FAIS. More specifically, the aim was to present trends for the PROMs used and which surgical approaches have been performed to treat patients with FAIS. The hypothesis was that an increase in the number of studies with arthroscopic procedures performed would be observed with the majority using hip specific PROMs.

## Methods

The systematic review was governed in agreement with the Preferred Reporting Items for Systematic Review and Meta-Analysis protocols (PRISMA) [142].

### Eligibility criteria

All inclusion and exclusion criteria were prespecified and designed as recommended by PRISMA. The inclusion criteria for this systematic review were clinical studies with patients undergoing surgical treatment for FAIS. Studies defined as prospective, retrospective and randomized controlled trials (RCTs) were included. Only studies comprising PROMs were included. The study could be either therapeutic or prognostic. Therapeutic studies defined as studies exploring the results of FAIS surgery, and, prognostic studies, defined as investigating the effect of a patients’ characteristic on the outcome of FAIS. Only studies with English language in full text were included.

Exclusion criteria were studies including adolescents, children or described as “open physes”. No studies with patients < 18 years were included. Studies with less than 8 patients were deemed not eligible. Studies with primarily patients with slipped capital femoral epiphysis and Leg-Calve-Perthes disease were excluded. Studies with radiographic measurements as only outcomes were also excluded. Conference papers, systematic reviews, commentaries, protocols, narratives and studies validating PROMs were excluded. Studies with primary purpose to evaluate other diagnoses than FAIS and studies with patients undergoing revision surgery were also excluded.

### Information sources and search

A systematic literature search was conducted in the online databases PubMed and Embase in September 2020. The searches were performed by a librarian with expertise in electronical searches at the Sahlgrenska University Hospital Library, Gothenburg, Sweden. The search retrieved studies from the period January 1999 until search day 7^th^ of September 2020 to include an interval of over 20 years. The search was performed with controlled terminology and words. Different variations of the terms for “*femoroacetabular impingement*” OR “*FAI”* OR “*hip impingement”* OR *“CAM impingement”* OR *“Pincer Impingement”* were used together with different variations of “*surgery*” OR *“operative”* OR *“arthroscopy”* to create the search string. Exact information about the details on the search strategies for the database PubMed is found in Appendix, (Table 2).

### Study selection

The studies from the electronic search were systematically evaluated by titles, thereafter abstract and finally their full texts by two reviewers (IL and SN). Both reviewers evaluated all studies from both databases independent of each other. Duplicates were removed manually. If the title or the abstract did not provide enough information regarding inclusion, the study was automatically included to the full-text assessment. The two reviewers were not blinded to the author, year and journal of publication. After all full texts were independently decided by the two reviewers, any disagreements regarding inclusion of studies were solved with discussion between the two reviewers.

### Data items

The data extracted included the level of evidence, title of the study, authors, year of publication, journal, country where study was performed, type of study (retrospective, prospective, RCT), included number of, and which different PROMs used in the study. The proportion of “hip specific” PROMs in the study was recorded in the extraction sheet. In addition to exploring the development of included PROMs over the years, 2016, when the Warwick agreement was stated, was used as a cut-off to evaluate the adoption of recommended PROMs. It was noted if the study had included any type of “rate of return to sport” (RTS) apart from using a regular PROM and if the study evaluated patient satisfaction. Inclusion of any RTS assessment was in this study defined dichotomously (yes or no). Type of interventions assessed in the study were divided into open, arthroscopic or a combination of arthroscopic/open. Further data as proportion of sex, follow-up time, and number of patients were collected. The number of patients were defined as the patients undergoing surgical intervention, i.e., if the control group consisted of patients without receiving intervention, the control group was not included. Distribution of sex and mean follow-up for the last visit were recorded.

### Statistical analyses

Interobserver agreement for full-texts was calculated with the Cohen kappa coefficient (κ) [119]. According to previous recommendations the values of κ were set a priori with a κ of 0–0.2 equals slight agreement, 0.21–0.4 fair agreement, 0.41–0.6 moderate agreement, 0.61–0.8 substantial agreement and > 0.8 equals to near perfect agreement. Descriptive statistics were used to present the data. Mean, standard deviation (SD), median and range values were presented when appropriate. Follow-up period was presented either as average follow-up period, or if not presented in the study, as minimum follow-up period. For studies comparing two or more groups, and no average follow-up period was mentioned for the entire cohort, a combined average follow-up was calculated. The analyses were performed with Microsoft Excel (version 16.40, Microsoft Corporation).

## Results

### Study identification and characteristics

**Fig. 1:**
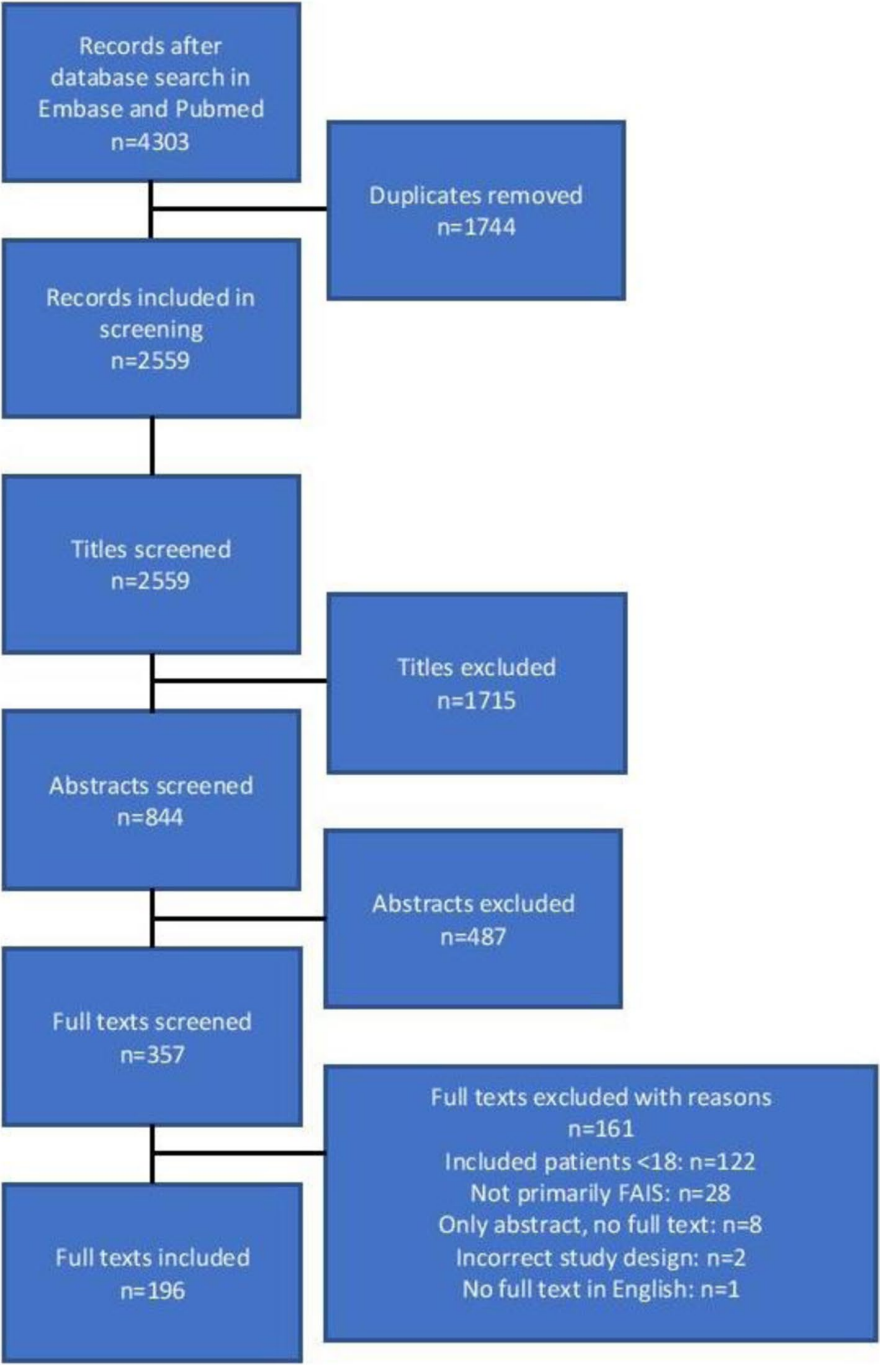
Flow chart of the screening process and number of included studies

The first search revealed 2,085 studies in PubMed and 2,218 studies in Embase. After removing duplicates, a total of 2,559 unique studies were eligible for the screening process. Figure 1 displays a flowchart of the screening process in accordance with the PRISMA guidelines. The agreement between the two readers for inclusion of full-text was 97% with a Cohen kappa value of 0.82, considered as near perfect agreement.

**Fig. 2:**
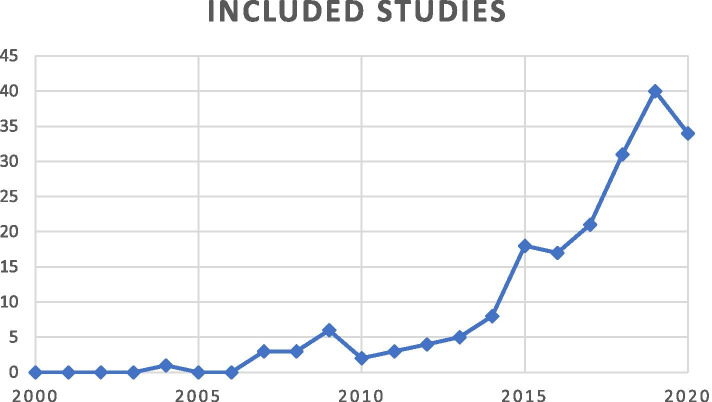
Trend over the years of included studies. *Note the year 2020 only covers studies until search day 7th of September

There were 6 (3%) RCTs, 55 (28%) prospective studies and 135 (69%) retrospective studies included in this systematic review. There were 6 (3%) Level I studies, 21 (11%) Level II studies, 86 (44%) Level III studies and 83 (42%) Level IV studies (Table 1). The included studies were published between 2004–2020. There was a large increase of published studies in the latter years where 143 (73%) of the studies were published in the last 5 years (2016–2020) compared to 7 (4%) in the first 5 years (2004–2008), an increase of 2,043% (Fig. 2).

**Table 1:** Included and results of individual studies

Author	Year	Level of evidence	Country	Study type	Follow-up	RTS	Participants	Included PROMs	Hip specific PROMS	Men%	Surgery
Abrahamson, J. [1]	2020	III	Sweden	Retrospective	23.4	y	551	HAGOS, iHOT-12, HSAS	3	77	ARTHROSCOPIC
Aguilera-Bohórquez, B. [2]	2020	IV	Colombia	Retrospective	12	n	17	WOMAC	1	47	ARTHROSCOPIC
Atzmon, R. [3]	2019	III	Israel	Retrospective	50^a^	n	64	HOS, mHHS, satisfaction	2	74	ARTHROSCOPIC
Avnieli, I. B. [4]	2020	III	Israel	Retrospective	24	y	133	HOS, mHHS, VAS satisfaction	2	62	ARTHROSCOPIC
Balazs, G. C. [5]	2018	II	USA	Prospective	1.5	n	59	HAGOS, iHOT-33, PCS, VAS pain	2	54	ARTHROSCOPIC
Barastegui, D. [6]	2018	IV	Spain	Retrospective	24	y	21	HOS (ADL + SS), mHHS, VAS pain	2	100	ARTHROSCOPIC
Bardakos, N. V. [7]	2008	III	England	Retrospective	12	n	71	mHHS	1	58	ARTHROSCOPIC
Basques, B. A. [8]	2019	III	USA	Retrospective	24	n	624	HOS (ADL + SS), mHHS, VAS pain, VAS satisfaction	2	35	ARTHROSCOPIC
Beaulé, P. E[10]	2017	IV	Canada	Prospective	24.5	n	10	HOOS	1	100	ARTHROSCOPIC
Beaulé, P. E. [9]	2007	IV	Canada	Retrospective	36	y	34	SF-12, UCLA, WOMAC	1	53	OPEN
Beck, E. C. [12]	2019	III	USA	Retrospective	32.9	n	108	HOS (ADL + SS), mHHS, VAS pain, VAS satisfaction	2	x	ARTHROSCOPIC
Beck, E. C. [14]	2020	III	USA	Retrospective	24	n	249	HOS (ADL + SS), iHOT-12, mHHS, VAS pain, VAS satisfaction	3	35	ARTHROSCOPIC
Beck, E. C. [16]	2020	IV	USA	Prospective	6	n	74	HOS (ADL + SS), iHOT-12	2	23	ARTHROSCOPIC
Beck, E. C. [17]	2020	III	USA	Retrospective	24	n	647	HOS (ADL + SS), iHOT-12, mHHS, VAS pain, VAS satisfaction	3	24	ARTHROSCOPIC
Beck, E. C. [15]	2020	III	USA	Retrospective	24	n	384	HOS (ADL + SS), mHHS, VAS pain, VAS satisfaction	2	32	ARTHROSCOPIC
Beck, E. C. [11]	2020	III	USA	Retrospective	50	n	264	HOS (ADL + SS), mHHS, VAS pain, VAS satisfaction	2	34	ARTHROSCOPIC
Beck, E. C. [13]	2019	III	USA	Retrospective	24	n	336	HOS (ADL + SS), iHOT-12, mHHS, VAS pain, VAS satisfaction	3	30	ARTHROSCOPIC
Beck, M. [18]	2004	IV	Switzerland	Retrospective	56.4	n	19	The Merle d'Aubigné and Postel hip score	1	74	OPEN
Bennett, A. N. [19]	2016	IV	England	Prospective	12	n	101	FAA, NAHS, VAS pain	1	75	ARTHROSCOPIC
Bolia, I. K. [20]	2019	III	USA	Retrospective	80^a^	n	126	HOS (ADL + SS), mHHS, SF-12, VAS satisfaction	2	57	ARTHROSCOPIC
Boone, G. R. [21]	2012	IV	USA	Retrospective	45.6	n	21	UCLA	0	64	OPEN
Briggs, K. K. [22]	2019	III	USA	Retrospective	61.2	n	230	HOS (ADL + SS), mHHS, SF12, VAS satisfaction, WOMAC, Tegner	3	x	ARTHROSCOPIC
Bryan, A. J. [23]	2016	III	USA	Retrospective	24	n	201	HOS (ADL + SS), mHHS	2	69	ARTHROSCOPIC
Byrd, J.W. [24]	2009	IV	USA	Prospective	16	n	207	mHHS	1	67	ARTHROSCOPIC
Byrd, J. W. [25]	2016	III	USA	Retrospective	37^a^	n	108	mHHS	1	52	ARTHROSCOPIC
Byrd, J. W. [26]	2019	III	USA	Retrospective	18.9	n	42	iHOT, mHHS	2	52	ARTHROSCOPIC
Campoamor González, M. [27]	2020	III	Spain	Retrospective	6	n	57	HHS	1	68	INCLUDING BOTH
Cancienne, J. [28]	2019	III	USA	Retrospective	24	n	1102	HOS (ADL + SS), mHHS, VAS pain, VAS satisfaction	2	35	ARTHROSCOPIC
Carreira, D. S. [29]	2018	IV	USA	Prospective	12	n	45	HOS (ADL + SS), mHHS, iHOT-12, SF-12	3	36	ARTHROSCOPIC
Casartelli N. [30]	2014	IV	Switzerland	Prospective	30	y	8	HOS (ADL + SS), satisfaction (1–5), pain change (1–5)	1	38	ARTHROSCOPIC
Catelli, D. S. [31]	2019	II	Canada	Prospective	24	n	11	HOOS	1	100	INCLUDING BOTH
Catelli, D. S. [32]	2019	II	Canada	Prospective	24	n	11	HOOS	1	100	INCLUDING BOTH
Cetinkaya, S. [33]	2016	III	Turkey	Retrospective	45.2	n	67	HOS, VAS pain	1	57	ARTHROSCOPIC
Chaharbakhshi, E. O. [34]	2019	III	USA	Retrospective	47^a^	n	107	HOS (SS), iHOT-12, mHHS, NAHS, VAS pain, VAS satisfaction	4	66	ARTHROSCOPIC
Chahla, J. [36]	2019	III	USA	Retrospective	27.8	n	634	HOS (ADL + SS), mHHS, VAS pain, VAS satisfaction	2	33	ARTHROSCOPIC
Chahla, J. [37]	2019	III	USA	Retrospective	24	n	600	HOS (ADL + SS), mHHS, VAS pain, VAS satisfaction	2	36	ARTHROSCOPIC
Chahla, J. [35]	2019	iii	USA	Prospective	12	n	153	HOS (ADL + SS), HPSES, mHHS, VAS pain, VAS satisfaction	3	29	ARTHROSCOPIC
Chambers, C. C. [38]	2019	IV	USA	Retrospective	24	n	142	HOOS, mHHS, SF-12, VAS pain	2	51	ARTHROSCOPIC
Chiron, P. [39]	2012	IV	France	Prospective	26.4	y	108	HHS, MOS, NAHS, SF-36, satisfaction (1–5), VAS pain, WOMAC	3	85	MINIMALLY INVASIVE APPROACH
Chladek, P. [40]	2015	III	Czech Republic	Retrospective	40	n	100	NAHS, WOMAC	2	x	MINI-INVASIVE SURGERY AND OPEN
Cho, S. H. [41]	2015	IV	Korea	Retrospective	24	n	11	mHHS, UCLA	1	36	ANTERIOR MINI-OPEN (AMO) AND OPEN
Christensen, J. C. [43]	2019	III	USA	Retrospective	24	n	173	iHOT-12	1	0	ARTHROSCOPIC
clapp, I. M. [44]	2020	II	USA	Prospective	19.9	n	85	HOS (ADL + SS), mHHS, iHOT-12, PCS, TSK, VAS pain, VAS satisfaction,	3	25	ARTHROSCOPIC
Claßen, T. [45]	2016	II	Germany	Prospective	6	n	177	NAHS, WOMAC	2	46	ARTHROSCOPIC
Comba, F. [47]	2016	IV	Argentina	Prospective	91	n	42	mHHS, WOMAC	2	64	ARTHROSCOPIC
Cunningham, D. J. [48]	2017	II	USA	Prospective	1.5	n	62	iHOT-12, PCS, PHQ, VAS pain	1	33	ARTHROSCOPIC
Cvetanovich, G. L. [49]	2017	III	USA	Retrospective	31.2	n	348	HOS (ADL + SS), mHHS, VAS pain, VAS satisfaction	2	42	ARTHROSCOPIC
Cvetanovich, G. L. [50]	2018	IV	USA	Prospective	24	n	386	HOS (ADL + SS), mHHS, VAS pain	2	39	ARTHROSCOPIC
Di Benedetto, P. [51]	2016	II	Italy	Prospective	12	n	65	mHHS, MHOT	2	x	ARTHROSCOPIC
Domb, B. G. [55]	2013	II	USA	Prospective	25.2	n	30	HOS (ADL + SS), mHHS, NAHS, VAS pain, VAS satisfaction	3	20	INCLUDING BOTH
Domb, B. G. [52]	2018	III	USA	Retrospective	50	n	130	HOS (SS), mHHS, NAHS, VAS pain, VAS satisfaction	3	28	ARTHROSCOPIC
Domb, B. G. [54]	2020	III	USA	Retrospective	24	n	148	HOS (SS), iHOT-12, mHHS, NAHS, SF-12, VAS pain, VAS satisfaction, VR-12	4	41	ARTHROSCOPIC
Domb, B. G. [53]	2014	III	USA	Retrospective	24	n	33	HOS (ADL + SS), mHHS, NAHS, VAS pain, VAS satisfaction	3	64	ARTHROSCOPIC
Drager, J. [56]	2020	III	USA	Retrospective	12	n	346	HOS (ADL + SS), iHOT-12, mHHS, VAS pain, VAS satisfaction	3	28	ARTHROSCOPIC
Ellis, S. H. [57]	2020	iii	Australia	Retrospective	12	n	79	iHOT-33	1	42	ARTHROSCOPIC
Ernat, J. J. [59]	2019	IV	USA	Retrospective	12	n	182	mHHS, SANE, satisfaction score, VAS pain, VR-12, WOMAC	2	74	MINI-OPEN ARTHROSCOPIC-ASSISTED
Ernat, J. J. [58]	2015	IV	USA	Retrospective	43.2	n	93	mHHS, SANE, satisfaction, VAS pain, VR-12, WOMAC	2	70	MINI-OPEN ARTHROSCOPIC-ASSISTED
Espinosa, N. [60]	2007	III	Switzerland	Retrospective	24	n	52	The Merle d’Aubigne´-Postel score	1	x	OPEN
Essilfie, A. A. [61]	2020	II	USA	Prospective	24	n	126	mHHS, NAHS	2	67	ARTHROSCOPIC
Fabricant, P. D. [62]	2015	III	USA	Retrospective	21	n	243	HOS (ADL + SS), iHOT-33, mHHS	3	49	ARTHROSCOPIC
Ferro, F. P. [63]	2015	IV	USA	Retrospective	30	n	184	mHHS, SF-12, WOMAC	2	x	ARTHROSCOPIC
Fiorentino, G. [64]	2015	IV	Italy	Retrospective	36	n	38	mHHS, patient satisfaction	1	59	ARTHROSCOPIC
Flores, S. E. [65]	2018	II	USA	Prospective	12	n	58	HOOS, mHHS, SF-12, VAS pain	2	53	ARTHROSCOPIC
Flores, S. E. [66]	2020	II	USA	Prospective	24	n	131	HOOS, mHHS, SF-12, VAS pain	2	45	ARTHROSCOPIC
Flores, S. E. [67]	2018	II	USA	Prospective	12	n	122	HOOS, mHHS, SF-12, VAS pain	2	47	ARTHROSCOPIC
Foreman, S.C. [68]	2020	II	USA	Prospective	12	n	42	HOOS	1	64	ARTHROSCOPIC
Frank, R. M. [69]	2019	III	USA	Retrospective	31.2	y	330	HOS (ADL + SS), mHHS, VAS pain, VAS satisfaction	2	100	ARTHROSCOPIC
Frank, R. M. [71]	2018	IV	USA	Retrospective	31.1	y	59	HOS (ADL + SS), mHHS VAS pain, VAS satisfaction	2	38	ARTHROSCOPIC
Frank, R. M. [70]	2016	II	USA	Prospective	33.6	n	150	HOS (ADL + SS), mHHS, VAS satisfaction	2	50	ARTHROSCOPIC
Fukui, K. [73]	2015	IV	USA	Retrospective	42	n	28	HOS (ADL + SS), mHHS, SF-12, VAS satisfaction, WOMAC	3	57	ARTHROSCOPIC
Fukui. K. [72]	2015	IV	USA	Retrospective	40	n	100	HOS (ADL + SS), mHHS, SF-12, VAS satisfaction, WOMAC,	3	50	ARTHROSCOPIC
Gao, F. [75]	2020	IV	China	Prospective	24	n	27	iHOT-12, mHHS, VAS pain	2	56	ARTHROSCOPIC
Gicquel, T. [76]	2014	IV	France	Prospective	55.2	n	58	WOMAC, satisfaction (1–4)	1	63	ARTHROSCOPIC
Gigi, R. [77]	2016	III	Israel	Retrospective	30.4	n	106	HOS (ADL), mHHS	2	65	ARTHROSCOPIC
Grace, T. [78]	2018	IV	USA	Prospective	X	n	43	HOOS	1	58	ARTHROSCOPIC
Grace, T. [79]	2018	II	USA	Prospective	X	n	46	HOOS, VAS pain	1	59	ARTHROSCOPIC
Grant, L. F. [80]	2017	I	England	RCT	3	n	18	EQ-5D, NAHS	1	33	ARTHROSCOPIC
Graves, M. L. [81]	2009	IV	USA	Retrospective	38	n	46	The Merle d’Aubigne´-Postel score	1	54	OPEN
Griffin, D. R. [83]	2018	I	England	RCT	12	n	213	EQ-5D, iHOT-33, SF12, UCLA	1	58	ARTHROSCOPIC
Gupta, A. [86]	2014	IV	USA	Prospective	28.3	n	47	HOS (ADL + SS), mHHS, NAHS, VAS pain, VAS satisfaction	3	60	ARTHROSCOPIC
Gupta, A. [85]	2015	III	USA	Retrospective	23.1	n	680	HOS (ADL + SS), mHHS, NAHS, VAS pain, VAS satisfaction	3	33	ARTHROSCOPIC
Ha, Y. C. [87]	2020	IV	Corea	Retrospective	24	n	62	mHHS, UCLA, VAS pain, VAS satisfaction	1	90	ARTHROSCOPIC
Hamula, M. J. [88]	2020	III	USA	Retrospective	31.6	n	226	mHHS, NAHS	2	39	ARTHROSCOPIC
Haskel, J. D. [89]	2020	III	USA	Retrospective	24	n	149	mHHS, NAHS	2	25	ARTHROSCOPIC
Hassebrock, J. D. [90]	2019	III	USA	Retrospective	24	n	133	HOS (SS), iHOT-12, mHHS, NAHS, VAS pain, VAS satisfaction	4	47	ARTHROSCOPIC
Herrmann, S. J. [91]	2016	IV	Germany	Retrospective	32	n	79	HOS (ADL + SS)	1	62	ARTHROSCOPIC
Horisberger, M. [92]	2010	IV	Switzerland	Prospective	36	n	20	NAHS, VAS pain	1	80	ARTHROSCOPIC
Hwang, J. M. [94]	2019	IV	Korea	Retrospective	43.6	n	9	HOS (ADL), mHHS, VAS pain	2	75	ARTHROSCOPIC
Ilizaliturri, V. M. [95]	2008	IV	Mexico	Prospective	24	n	19	WOMAC	1	58	ARTHROSCOPIC
İnan, U. [96]	2016	IV	Turkey	Retrospective	48	n	21	HHS	1	33	OPEN
Ishøi, L. [97]	2018	III	Denmark	Retrospective	33.1	y	189	HAGOS	1	51	ARTHROSCOPIC
Ishøi, L. [98]	2019	III	Denmark	Retrospective	33.1	y	184	HAGOS	1	50	ARTHROSCOPIC
Javed, A. [99]	2011	IV	England	Retrospective	30	n	40	mHHS, NAHS, satisfaction y/n	2	65	ARTHROSCOPIC
Jochimsen, K. N. [100]	2019	III	USA	Retrospective	X	n	127	HOOS	1	26	ARTHROSCOPIC
Jäger, M. [101]	2011	IV	Germany	Prospective	12	n	22	HHS	1	32	OPEN
Kaldau, N. C. [102]	2018	IV	Denmark	Retrospective	82.9^b^	n	84	EQ-5D, HAGOS, HSAS	2	54	ARTHROSCOPIC
Kaplan, D. J. [103]	2020	IV	USA	Retrospective	76.5	n	103	HHS, mHHS, NAHS	3	32	ARTHROSCOPIC
Keating, T. C. [104]	2019	IV	USA	Retrospective	24	y	22	HOS (ADL + SS), mHHS, VAS pain, VAS satisfaction	2	0	ARTHROSCOPIC
Kekatpure, A. L. [105]	2017	III	Korea	Retrospective	25.4	n	83	mHHS, NAHS, WOMAC	3	66	ARTHROSCOPIC
Kierkegaard, S. [107]	2020	II	Denmark	Prospective	12	y	60	HAGOS	1	37	ARTHROSCOPIC
Kierkegaard, S. [108]	2019	II	Denmark	Prospective	12	n	60	HAGOS	1	40	ARTHROSCOPIC
Kockara, N. [109]	2018	IV	Turkey	Retrospective	72	n	33	HHS	1	58	OPEN
Kouk, S. [110]	2020	III	USA	Retrospective	24	n	62	mHHS, NAHS	2	44	ARTHROSCOPIC
Krishnamoorthy, V. P. [112]	2019	III	USA	Retrospective	24	n	830	HOS (ADL + SS), iHOT-12, mHHS, VAS pain, VAS satisfaction	3	31	ARTHROSCOPIC
Krishnamoorthy, V. P. [111]	2019	III	USA	Retrospective	36.8	n	743	HOS (ADL + SS), mHHS, VAS pain, VAS satisfaction	2	32	ARTHROSCOPIC
Krych, A. J. [113]	2016	III	USA	Retrospective	24	n	104	HOS (ADL + SS), mHHS	2	38	ARTHROSCOPIC
Krych, A. J. [114]	2013	I	USA	RCT	32	n	36	HOS (ADL + SS)	1	0	ARTHROSCOPIC
Kunze, K. N. [115]	2019	III	USA	Retrospective	24	n	1094	HOS (ADL + SS), iHOT-12, mHHS, VAS pain, VAS satisfaction	3	34	ARTHROSCOPIC
Kunze, K. N. [116]	2019	III	USA	Retrospective	24	n	306	HOS (ADL + SS), mHHS, VAS pain, VAS satisfaction	2	42	ARTHROSCOPIC
Kunze, K. N. [117]	2019	IV	USA	Prospective	6	n	52	HOS (ADL + SS), iHOT-12, mHHS, PSQI, VAS pain	3	37	ARTHROSCOPIC
Lall, A. C. [118]	2020	III	USA	Retrospective	54.9	n	84	HOS (SS), iHOT-12, mHHS, NAHS, SF-12, VAS pain, VR-12	4	36	ARTHROSCOPIC
Lansdown, D. A. [120]	2018	IV	USA	Retrospective	24	n	707	HOS (ADL + SS), mHHS, VAS pain, VAS satisfaction	2	36	ARTHROSCOPIC
Lansdown, D. A. [121]	2018	III	USA	Retrospective	24	n	301	HOS (ADL + SS), mHHS, VAS pain, VAS satisfaction	2	36	ARTHROSCOPIC
Lee, S. [122]	2015	IV	USA	Retrospective	21	n	131	mHHS, VAS satisfaction	1	56	ARTHROSCOPIC
Lerch, S. [123]	2015	IV	Germany	Prospective	3.3	n	40	HOOS, WOMAC	2	x	ARTHROSCOPIC
Levy, D. M. [124]	2017	III	USA	Retrospective	24	n	84	HOS (ADL + SS), mHHS, VAS pain, VAS satisfaction	2	36	ARTHROSCOPIC
Lindman, I. [125]	2020	IV	Sweden	Prospective	60	n	64	HAGOS, HSAS, iHOT-12, VAS hip function, EQ-5D, EQ VAS, satisfaction y/n	3	81	ARTHROSCOPIC
Malagelada, F. [127]	2015	IV	Spain	Prospective	12	y	14	LISOH, VAS pain	1	64	MINI-OPEN TECHNIQUE
Maldonado, D. R. [128]	2020	III	USA	Retrospective	24	n	145	HOS (SS), iHOT-12, mHHS, NAHS, SF-12, VAS pain, VAS satisfaction, VR-12	4	12	ARTHROSCOPIC
Malloy, P. [129]	2019	IV	USA	Retrospective	26.4	n	50	HOS (ADL + SS), iHOT-12, mHHS, VAS pain, VAS satisfaction	3	36	ARTHROSCOPIC
Mannion, A. F. [130]	2013	II	Switzerland	Prospective	12	n	86	GTO, OHS, NASS	2	44	MINI-OPEN AND ARTHROSCOPIC
Mansell, N. S. [131]	2018	I	USA	RCT	12	n	40	GRC, HOS (ADL + SS), iHOT-33, PCS, Self-motivation inventory score, VAS pain	2	53	ARTHROSCOPIC
Mardones, R. [132]	2016	IV	Chile	Retrospective	52.8	n	23	mHHS, VAS pain	1	22	ARTHROSCOPIC
Mardones, R. [133]	2016	IV	Chile	Retrospective	48	n	15	mHHS, VAS pain, VHS	2	27	ARTHROSCOPIC
Martínez, D. [135]	2015	IV	Colombia	Retrospective	23.8	n	179	WOMAC	1	35	ARTHROSCOPIC
Mas Martinez, J. [136]	2020	IV	Spain	Retrospective	24	y	185	HOS (ADL + SS), iHOT-12 mHHS	3	77	ARTHROSCOPIC
Matsuda, D. K. [137]	2013	III	USA	Retrospective	30	n	54	NAHS, satisfaction scale	1	59	ARTHROSCOPIC
Matsuda, D. K. [138]	2017	III	USA	Retrospective	12	n	77	NAHS, satisfaction (1–5)	1	52	ARTHROSCOPIC
Matsuda, D. K. [139]	2019	III	USA	Retrospective	24	n	437	iHOT-12	1	67	ARTHROSCOPIC
Menge, T. J. [140]	2017	III	USA	Retrospective	120	n	154	HOS (ADL + SS), mHHS, SF-12, VAS satisfaction	2	52	ARTHROSCOPIC
Mladenović, D. [141]	2014	IV	Serbia	Retrospective	12	n	21	WOMAC	1	23	OPEN
Naal, F. D. [144]	2017	III	Switzerland	Retrospective	44.4	n	232	EQ-5D, EQ-VAS, OHS, satisfcation scale (1–5), UCLA	1	49	INCLUDING BOTH
Nabavi, A. [145]	2015	III	Australia	Retrospective	12	n	253	mHHS, NAHS	2	50	ARTHROSCOPIC
Nakashima, H. [146]	2019	III	Japan	Retrospective	34.1	n	97	mHHS, NAHS	2	44	ARTHROSCOPIC
Nawabi, D. H. [147]	2016	III	USA	Retrospective	24	n	177	HOS (ADL + SS), iHOT-33, mHHS	3	46	ARTHROSCOPIC
Nepple, J. J. [148]	2015	IV	USA	Prospective	X	n	50	mHHS, SF-12	1	64	ARTHROSCOPIC
Nepple, J. J. [149]	2009	III	USA	Retrospective	24^a^	n	48	mHHS	1	60	ARTHROSCOPIC AND LIMITED OPEN OSTEOCHONDROPLASIA
Nho, S. J. [150]	2019	III	USA	Retrospective	27.8	n	935	HOS (ADL + SS), iHOT-12, mHHS, VAS pain, VAS satisfaction	3	37	ARTHROSCOPIC
Nwachukwu, B. U. [151]	2020	III	USA	Retrospective	24	n	898	HOS (ADL + SS), mHHS, VAS pain, VAS satisfaction	2	35	ARTHROSCOPIC
Nwachukwu, B. U. [152]	2018	III	USA	Retrospective	24	n	719	HOS (ADL + SS), iHOT-33, mHHS	3	47	ARTHROSCOPIC
Nwachukwu, B. U. [153]	2017	III	USA	Retrospective	12	n	364	HOS (ADL + SS), iHOT-33, mHHS	3	43	ARTHROSCOPIC
Palmer, A. J. R. [156]	2019	I	England	RCT	8	n	112	EQ-5D, EQ-VAS, HADS (anxiety + depression), HAGOS, HOS (ADL + SS), iHOT-33, NAHS, OHS, Pain detect score, UCLA	5	34	ARTHROSCOPIC
Park, M. S. [157]	2014	IV	Korea	Retrospective	28.2	n	197	mHHS, VAS satisfaction	1	49	ARTHROSCOPIC
Perets, I. [160]	2019	III	USA	Retrospective	60	n	52	HOS (SS), iHOT-12, mHHS, NAHS, VAS pain, VAS satisfaction	4	72	ARTHROSCOPIC
Perets, I. [161]	2018	III	USA	Retrospective	71	n	148	HOS (SS), mHHS, NAHS, VAS pain, VAS satisfaction	3	39	ARTHROSCOPIC
Perets, I. [159]	2018	IV	USA	Retrospective	60	n	94	HOS (SS), mHHS, NAHS, VAS pain, VAS satisfaction	3	45	ARTHROSCOPIC
Philippon, M. J. [164]	2010	IV	USA	Retrospective	24	y	28	mHHS, VAS satisfaction	1	100	ARTHROSCOPIC
Philippon, M. J. [162]	2009	IV	USA	Prospective	27.6	n	112	HOS (ADL + SS), mHHS, NAHS, VAS satisfaction	3	45	ARTHROSCOPIC
Philippon, M. J. [163]	2012	IV	USA	Prospective	35.7	n	153	HOS (ADL + SS), mHHS, SF-12, VAS satisfaction	2	47	ARTHROSCOPIC
Polesello, G. C. [165]	2012	IV	Brazil	Retrospective	34.3	y	47	mHHS, satisfaction	1	43	ARTHROSCOPIC
Polesello, G. C. [166]	2009	IV	Brazil	Retrospective	27	n	28	HHS	1	67	ARTHROSCOPIC
Potter, M. Q. [167]	2014	II	USA	Prospective	X	n	147	HOS (ADL + SS), mHHS, Modified zung depression scale, MSPQ	2	37	ARTHROSCOPIC
Przybyl, M. [168]	2018	III	Poland	Retrospective	24	y	129	mHHS, NAHS	2	100	ARTHROSCOPIC
Ragab, R. [169]	2018	IV	Egypt	Prospective	12.5	n	40	iHOT-12, mHHS	2	50	ARTHROSCOPIC
Ramos, N. [171]	2020	III	USA	Retrospective	12	n	70	mHHS	1	47	ARTHROSCOPIC
Ramos, N. [172]	2020	IV	USA	Retrospective	19.2	y	10	mHHS, satisfaction	1	100	ARTHROSCOPIC
Redmond, J. M. [173]	2015	III	USA	Retrospective	24	n	190	HOS (ADL + SS), mHHS, NAHS, VAS pain, VAS satisfaction	3	37	ARTHROSCOPIC
Rego, P. A. [174]	2018	III	Portugal	Retrospective	59	y	198	NAHS	1	56	INCLUDING BOTH
Ribas, M. [176]	2007	IV	Spain	Retrospective	29.2	y	32	The Merle d’Aubigné -Postel score, WOMAC	2	72	MINI-OPEN TECHNIQUE
Riff, A. J. [177]	2018	IV	USA	Retrospective	24	y	32	HOS (ADL + SS), mHHS, VAS pain, VAS satisfaction	2	40	ARTHROSCOPIC
Rivera, E. [178]	2020	III	Spain	Retrospective	24	n	80	iHOT-33, mHHS, VAS pain	2	66	ARTHROSCOPIC
Roos, B. D. [179]	2017	III	Brazil	Retrospective	36^a^	n	56	mHHS, NAHS	2	84	INCLUDING BOTH
Roos, B. D. [180]	2015	IV	Brazil	Retrospective	29.1	n	40	mHHS, NAHS	2	87	ARTHROSCOPIC
Rylander, J. H. [181]	2011	IV	USA	Prospective	12	n	11	Tegner	0	73	ARTHROSCOPIC
Saltzman, B. M. [182]	2017	III	USA	Retrospective	31.2	n	381	HOS (ADL + SS), mHHS, VAS pain, VAS satisfaction	2	39	ARTHROSCOPIC
Samaan, M. A. [183]	2020	II	USA	Prospective	7	n	10	HOOS	1	80	ARTHROSCOPIC
Sanders, T. L. [184]	2017	IV	USA	Retrospective	30	y	46	ADL, IHOT, mHHS, sport score, subjective level of function (1–4)	2	33	ARTHROSCOPIC
Sansone, M. [186]	2015	IV	Sweden	Prospective	12.3	n	85	EQ-5D, HAGOS, HSAS, iHOT-12, VAS overall hip function, satisfaction y/n	3	80	ARTHROSCOPIC
Sansone, M. [187]	2016	IV	Sweden	Prospective	26	n	75	EQ-5D, HAGOS, HSAS, iHOT-12, VAS overall hip function, satisfaction y/n	3	77	ARTHROSCOPIC
Sansone, M. [188]	2017	IV	Sweden	Prospective	25.4	n	289	EQ-5D, HAGOS, HSAS, iHOT-12, VAS overall hip function, satisfaction y/n	3	66	ARTHROSCOPIC
Sariali, E. [189]	2018	IV	France	Prospective	39.6	n	47	HHS, OHS	2	x	ARTHROSCOPIC
Scanaliato, J. P. [190]	2018	III	USA	Retrospective	24	n	152	iHOT-12, mHHS, SF-12, VAS pain, VAS satisfaction	2	42	ARTHROSCOPIC
Shaw, K. A. [191]	2017	IV	USA	Prospective	6	n	11	HOS, mHHS	2	73	ARTHROSCOPIC
Shibata, K. R. [192]	2017	III	USA	Retrospective	18.9	y	98	HSAS, iHOT-33, mHHS	3	50	ARTHROSCOPIC
Skendzel, J. G. [194]	2014	III	USA	Retrospective	73	n	559	HOS (ADL + SS), mHHS, SF-12, VAS satisfaction, WOMAC	3	44	ARTHROSCOPIC
Skowronek, P. [195]	2017	IV	Poland	Retrospective	45	y	39	HHS, SF-36, VAS pain	1	64	MIN-OPEN DIRECT ANTERIOR APPROACH (DDA)
Sochacki, K. R. [198]	2018	III	USA	Retrospective	X	n	212	HOS (ADL + SS), iHOT-12, SF-36	2	44	ARTHROSCOPIC
Sochacki, K. R. [197]	2018	III	USA	Retrospective	12	n	77	BDI-2, HOS (ADL + SS), iHOT-33	2	27	ARTHROSCOPIC
Spencer-Gardner, L. [199]	2017	III	Australia	Retrospective	19	n	36	mHHS, NAHS	2	42	ARTHROSCOPIC
Srinivasan, S. C. [200]	2013	IV	England	Retrospective	22.3	n	26	NAHS, UCLA, VAS pain	2	42	COMBINED ARTHROSCOPIC AND OPEN
Stone, A. V. [201]	2019	IV	USA	Retrospective	24	n	626	HOS (SS), VAS pain, VAS satisfaction	1	31	ARTHROSCOPIC
Stone, A. V. [202]	2019	III	USA	Retrospective	24	n	688	HOS (ADL + SS), iHOT-12, mHHS, VAS pain, VAS satisfaction	3	35	ARTHROSCOPIC
Stähelin, L. [203]	2008	IV	Switzerland	Prospective	6	n	22	NAHS, VAS pain	1	68	ARTHROSCOPIC
Thomas, D. D. [204]	2017	IV	USA	Retrospective	30	n	469	SANE, VAS pain	0	66	ARTHROSCOPIC
Tjong, V. K. [207]	2016	IV	USA	Prospective	24	y	23	HOS (SS), iHOT-12, mHHS, VAS pain, VAS satisfaction	3	35	ARTHROSCOPIC
Vahedi, H. [208]	2019	III	USA	Retrospective	49.9	n	601	mHHS, SF-36	1	54	ARTHROSCOPIC
Wadhwani, J. [209]	2018	IV	Spain	Retrospective	12	n	105	mHHS	1	50	ARTHROSCOPIC
Westermann, R. W. [210]	2018	III	USA	Retrospective	X	n	321	HOOS (pain + physical function), UCLA, VR-12	1	31	ARTHROSCOPIC
Wu, C. T. [211]	2019	IV	Taiwan	Retrospective	44	n	36	HHS, VAS pain	1	56	MINI-OPEN ARTHROSCOPIC-ASSISTED
Wörner, T. [212]	2019	III	Sweden	Retrospective	8.1	y	33	HAGOS, HSAS	2	88	ARTHROSCOPIC
Yoo, J. I. [214]	2017	IV	Korea	Retrospective	24	n	40	mHHS, UCLA, VAS pain	1	63	ARTHROSCOPIC
Yun, H. H. [215]	2009	IV	Korea	Retrospective	27.6	n	16	HHS	1	86	OPEN
Zhu, X. [217]	2020	I	China	RCT	3	n	100	HHS, PGA, VAS pain	1	51	ARTHROSCOPIC
Zimmerer, A. [218]	2018	II	Germany	Prospective	24.4	n	43	HOOS, WOMAC	2	72	ARTHROSCOPIC
Zusmanovich, M. [219]	2020	III	USA	Retrospective	25.2	n	34	mHHS, NAHS, VAS pain	2	41	ARTHROSCOPIC
Öhlin, A. [220]	2017	IV	Sweden	Prospective	24	n	198	iHOT-12, satisfaction y/n	1	62	ARTHROSCOPIC

*Abbreviations*: *n* no, *PROM* Patient-reported Outcome Measures, *RCT* randomized control trial, *RTS* Return to sport, *l y* = yes. For abbrevaiations of PROMs, see Appendix, Table 3

^a^combined mean value was calculated

^b^median value

More than half of the studies (58%) were conducted in USA. Most studies were published in *The American Journal of Sports Medicine* (21%), followed by *Arthroscopy: The Journal of Arthroscopic and Related Surgery* (19%). A total of 32,303 patients were included counting the patients in all studies together, with an average of 165 patients per study (range 8–1,102). The mean follow-up period was 27.0 months (± 17 SD), (range 1.5–120) (Table 1).

### Surgical procedure

The majority of the included studies (85%) were evaluating arthroscopic treatment. Only 5% of the included studies were examining solely open dislocation while the remaining 10% discussed either both open and arthroscopic or defined a mini-open technique with arthroscopic assistance. The procedure described in each study is reported in Table 1.

### Patient-reported outcome measures

A total of 39 different PROMs were found in the studies, of these, 15 (38%) were hip-specific (Table 3, in Appendix). Between 1–10 PROMs were used in each study with an average of 3 (± 1.8 SD) PROMs per study. Before 2016, the median of included PROMs was two per study, and after 2016 the median had increased to three per study.

**Fig. 3:**
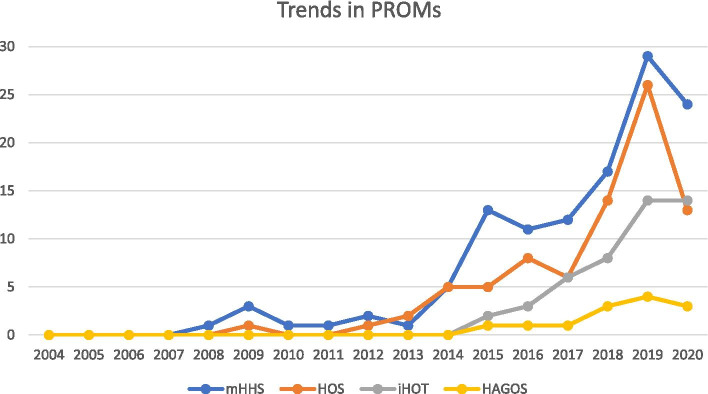
Trends in the number of recommended PROMs for FAIS and the most commonly used mHHS Abbreviations: HAGOS: The Copenhagen Hip and Groin Outcome Score, HOS: Hip Outcome Score, iHOT: international Hip Outcome Tool, mHHS: modified Harris Hip score, PROM: Patient-reported Outcome Measure. *Note the year 2020 only covers studies until search day 7^th^ of September

The most common used hip-specific PROM was mHHS (used in 120 studies (61%)), followed by HOS (81 studies (41%)) (Fig. 3). An additional question of return to sport/return to activity was seen in 13% of the included studies. Of 196 studies, 40% included a question on satisfaction of which the majority used the visual analog scale.

During the first five years (2004–2008), the Merle d’Aubigné and Postel score and the Western Ontario and McMaster Universities Osteoarthritis Index (WOMAC) were equally the most commonly used scores, reported in 3 (43%) of the studies during that period. During the last five years (2016–2020), the mHHS was the most commonly used, in 93 (65%) of the studies.

Of the 143 studies published during or after 2016, 67 (47%) studies have included the HOS, 46 (32%) included either iHOT-12 or iHOT-33 and 12 (8%) studies included the HAGOS (Fig. 3). Fifty-two of the 143 studies (36%) did not use any of the three PROMs recommended by the Warwick agreement [82] (Table 1).

## Discussion

The most important finding in this systematic review was the expected growth in the number of studies published over the years, where over 70% of the included studies were published between 2016–2020. Although the literature review included studies from 1999–2020, the first study meeting the inclusion criteria was published in 2004.

A total of 39 different PROMs were used among the studies, of which 15 were hip specific. The most common non-hip specific outcome was satisfaction, found in 40% of the studies. Previous studies have reported that satisfaction is the most frequently used non-hip specific outcome tool, although there is a variability how satisfaction is reported [175, 193]. The discrepancy in the use of different PROMs has previously been noted and the reason for this is unknown. The routinely use of a specific PROM, the difficulty in changing PROMs once norms have been established and the inevitable retention of the same PROMs to be able to follow a cohort and evaluate long-term outcomes are possible explanations for the divergence in use of PROMs [175].

After the Warwick agreement in 2016, three patient-reported outcome measures were considered suitable for the target population of FAIS and were recommended to use when evaluating surgery for FAIS [82], 65% of the included studies in this systematic review used at least one of the recommended PROMs (HAGOS, iHOT-12 or iHOT-33 and HOS (ADL + SS)). Nonetheless, the mHHS remains being the most commonly used PROM, even though there is a well-known ceiling effect of mHHS described for young active patients [206]. It could be seen as both surprising and concerning that mHHS still is the most used PROM in studies on FAIS as its outcome’s validity for young and active patients is considered low. Thorborg et al. [206] found HAGOS to be the best suited PROM for patients with FAIS, which only was used in 7% of the studies. This finding can guide future healthcare providers and researchers in using hip specific PROMs valid for the target population and diagnosis. Furthermore, there is a need for adoption of new validated scores, translated into the patients’ native language.

Only 13% of the included studies reported RTS specifically by using a clear definition. There is a current challenge in sports science regarding the definition of RTS, and the most optimal evaluation of RTS has not yet been decided. Activity scores such as the HOS (SS), Tegner activity scale or HSAS, with the purpose to evaluate the patients’ activity level or issues in sport specific activities, are not the best tools to evaluate the RTS. Mainly because these scores do not include training load or performance compared with preinjury status. This could possibly generate a ceiling effect if the patients rate the PROMs higher, yet still not being capable to fully return to their preinjury level of sport. Furthermore, the definition of RTS has been proposed to differ between elite and recreational athletes [42]. Athletes undergoing hip arthroscopic surgery for FAIS usually have a major interest whether they can RTS again, thus, a reliable method to determine RTS is thus needed.

The majority of the studies were published in USA or in Europe. This has previously been reported [106, 213]. Although USA and Europe have been in the front line of hip arthroscopic surgery and research, a small number of studies included in this systematic review were from Korea and China, indirectly indicating an upcoming trend in performed surgeries for FAIS in Asia. Moreover, only studies in the English language were included in this systematic review, which partly might explain the high percentage of studies from USA and Europe.

Although a few RCT:s have been published, retrospective studies are still the most common. Over the years, patient registries have facilitated prospective evaluation of FAIS and yielded important insight on PROMs [126, 185]. Öhlin et al. [155] assessed the methodological quality of prospective studies over a 5-year time period and found no improvement in the quality of the methods despite an increase in the number of published studies. With the dramatic increase seen in the number of published studies in this systematic review, it is of importance to also improve the quality of observational studies. New consensus meetings to enhance adoption of suitable PROMs and education of researchers and clinicians could benefit future research in the outcome of FAIS.

### Strengths and limitations

The strength of this study is the methodological rigor using PRISMA guidelines, focus on an important topic and the longitudinal analysis of a 20-year time horizon.

This systematic review is not without limitations. One of the a-priori set exclusion criteria was age, excluding studies with patients < 18 years old, though the focus was on the adult population as validation of PROMS in the pediatric population is still emerging. Moreover, only publications in the English language were included and there is a risk of missing publications in non-English speaking countries. Due to the heterogeneity of the included studies no statistical meta-analysis was conducted.

## Conclusion

There has been a continuous increase in the number of published studies regarding FAIS with the majority evaluating arthroscopic surgery. The mHHS remains being the most commonly used PROM.

## Data Availability

All data analyzed is included in the published study and its supplementary information files or references.

## Appendix

**Table 2:** Search strategy: pubmed^a^

Search	Query	Results
#27	Search: #19 NOT #22 Filters: English Sort by: Most Recent	2,085
#23	Search: #19 NOT #22 Sort by: Most Recent	2,172
#22	Search: #20 OR #21 Sort by: Most Recent	5,073,653
#21	Search: animal[ti] OR animals[ti] OR rat[ti] OR rats[ti] OR mouse[ti] OR mice[ti] OR rodent[ti] OR rodents[ti] OR dog[ti] OR dogs[ti] OR cat[ti] OR cats[ti] OR koalas[ti] OR hamster[ti] OR hamsters[ti] OR rabbit[ti] OR rabbits[ti] OR swine[ti] OR murine[ti] Sort by: Most Recent	1,886,518
#20	Search: ((animals[mh]) NOT (animals[mh] AND humans[mh])) Sort by: Most Recent	4,731,731
#19	Search: #5 AND #18 Sort by: Most Recent	2,177
#18	Search: #6 OR #7 OR #17 Sort by: Most Recent	2,006,557
#17	Search: surgery[tiab] OR surgical[tiab] OR operative[tiab] OR minimally invasive[tiab] Sort by: Most Recent	1,989,360
#7	Search: arthroscop*[tiab] Sort by: Most Recent	31,803
#6	Search: "Arthroscopy"[Mesh] Sort by: Most Recent	23,951
#5	Search: #2 OR #3 OR #4 Sort by: Most Recent	4,313
#4	Search: hip impingement[tiab] OR cam impingement[tiab] OR pincer impingement[tiab] OR FAI[tiab] OR FAIS[tiab] Sort by: Most Recent	2,865
#3	Search: (femoroacetabular[tiab] OR femoracetabular[tiab] OR femoral acetabular[tiab] OR femoro-acetabular[tiab]) AND impingement[tiab] Sort by: Most Recent	2,738
#2	Search: "Femoracetabular Impingement"[Mesh] Sort by: Most Recent	1,702

^a^Date of search: 7th of September 2020. Results: 2085 studies

**Table 3:** Included patient-reported outcome measures (PROMs) and their abbreviations

PROM	Name	Hip specific
BDI-2	Beck Depression Inventory	No
EQ-5D	European Quality of life index version 5D	No
FAA	Functional Activity Assessment	No
GRC	Global Rating of Change	No
GTO	Global Treatment Outcome	No
HADS	Hospital Anxiety and Depression Scale	No
HAGOS	The Copenhagen Hip and Groin Outcome Score	Yes
HHS	Harris Hip Score	Yes
HOOS	Hip Disability and Osteoarthritis Outcome Score	Yes
HOS (ADL + SS)	Hip Outcome Score (Activities of Daily Living + Sport Specific)	Yes
HPSES	Hip Preservation Surgery Expectations Survey	Yes
iHOT-12	The international Hip Outcome Tool-12	Yes
iHOT-33	The international Hip Outcome Tool-33	Yes
LISHO	Lequesne Functional Index for Hip Osteoarthritis	Yes
Merle d'Aubigne and Postel scale		Yes
mHHS	modified Harris Hip Score	Yes
MHOT	Mahorn Hip Outcome Tool	Yes
MSPQ	Modified Somatic Perception Questionnaire	No
Modified zung depression scale	-	No
NASS	North American Spine Society Lumbar Spine Questionnaire	No
MOS	Mean Opinion Score	No
NAHS	Non-Arthritic Hip Score	Yes
OHS	Oxford Hip Score	Yes
Pain detect score	-	No
PCS	Pain Catastrophizing Scale	No
PGA	Patient Global Assessment	No
PHQ	Patient Health Questionnaire	No
PSQI	Pittsburgh Sleep Quality Index	No
SANE	Single Assessment Numeric Evaluation	No
Satisfaction		No
SF-12	12-item Short-Form Health Survey	No
SF-36	The Short Form 36 Health Survey	No
Tegner	-	No
TSK	Tampa Scale of Kinesiophobia	No
UCLA	University of California Los Angeles activity scores.	No
VAS pain	Visual analoge scale	No
VHS	Vail Hip score	Yes
VR-12	The Veterans RAND 12 Item Health Survey	No
WOMAC	Western Ontario and MacMaster Universities Osteoarthritis Index	Yes

## Abbreviations

FAIS: Femoroacetabular impingement syndrome
HAGOS: Hip and Groin outcome score
iHOT: International Hip Outcome Tool
mHHS: Modified Harris Hip Score
PRISMA: Preferred Reporting Items for Systematic Review and Meta-Analysis
PROM: Patient-reported outcome measure
RCT: Randmoized controlled trial
RTS: Return to sports
SD: Standard Deviation
WOMAC: Western Ontario and McMaster Universities Osteoarthritis Index
